# Development and validation of a new patient experience tool in patients with serious illness

**DOI:** 10.1186/s12904-016-0172-x

**Published:** 2016-12-30

**Authors:** Karl M. Fernstrom, Nathan D. Shippee, Alissa L. Jones, Heather R. Britt

**Affiliations:** 1 Division of Applied Research, Allina Health, 2925 Chicago Avenue, Mail Stop #10039, Minneapolis, MN 55407 USA; 2 Division of Health Policy and Management, University of Minnesota School of Public Health, 420 Delaware St. SE, D375 Mayo MMC 729, Minneapolis, MN 55455 USA

**Keywords:** Patient experience, Chronic disease, Health care surveys, Patient-centered care, Patient-reported outcome measures

## Abstract

**Background:**

Patients with serious chronic illnesses face increasingly complex care and are at risk of poor experience due to a fragmented health system. Most current patient experience tools are not designed to address the unique care aspects of this population and the few that exist are delivered too late in the disease trajectory and are not administered longitudinally which makes them less useful across settings.

**Methods:**

We developed a new tool designed to address these gaps. The 25 item scale was tested and refined using randomly cross-validated exploratory and confirmatory factor analyses. Participants were not yet hospice eligible but sick enough to receive benefits of a supportive care approach in the last 2 to 3 years of life. Full information maximum likelihood models were run to confirm the factor structure developed in exploratory analyses. Goodness-of-fit was assessed with the Comparative Fit Index, the Tucker-Lewis Index, and the Root Mean Square Error of Approximation. Test-retest reliability was assessed with the intraclass correlation coefficient and internal consistency of the final scale was examined using Cronbach’s alpha.

**Results:**

Exploratory factor analysis revealed three domains — Care Team, Communication, and Care Goals — after removing weak loading and cross loading items. The initial three domain measurement model suggested in the development cohort was tested in the validation cohort and exhibited poor fit *X* ^2^ (206) = 565.37, *p* < 0.001; CFI = 0.879; TLI = 0.864; RMSEA = 0.076. After model respecification, including removing one additional item and allowing paths between theoretically plausible error terms, the final 21 item tool exhibited good fit *X* ^2^ (173) = 295.63, *p* < 0.001; CFI = 0.958; TLI = 0.949; RMSEA = 0.048. Cronbach’s alpha revealed high reliability of each domain (Care Team = 0.92, Communication = 0.83, Care Goals = 0.77) and the entire scale (α = 0.91). ICC showed adequate test-retest validity (ICC = 0.58; 95% CI: 0.52–0.65) of the full scale.

**Conclusions:**

When administered earlier in the chronic illness trajectory, a new patient experience scale focused on care teams across settings, communication, and care goals, displayed strong reliability and performed well psychometrically.

**Trial registrations:**

This trial (NCT01746446) was registered at ClinicalTrials.gov on November 27, 2012 (retrospectively registered).

**Electronic supplementary material:**

The online version of this article (doi:10.1186/s12904-016-0172-x) contains supplementary material, which is available to authorized users.

## Background

The Commonwealth Fund has stated that health care expenditures in the United States (U.S.) are far higher than those of other developed countries yet our results are not better [1]. Berwick et al. laid the political foundations for improving upon the U.S. health care system through the pursuit of three aims — commonly referred to as the Triple Aim [2] of: improving the experience of care, improving the health of populations, and reducing costs. With the signing of the Affordable Care Act (ACA) in the fall of 2010 [3], health systems in the U.S. are now incentivized to deliver improved patient experience by the Centers for Medicare & Medicaid Services (CMS) through its value-based purchasing program [4]. As the single largest payer for health care in the U.S., CMS is positioned well to drive change nationally. Penalties went into effect in the fall of 2012 for inpatients and will go into effect on all CMS patients by 2018. Health systems in the U.S. are faced with a need to improve the care experience for their patients. In order to do so, providers must be able to track and understand the experiences of some of their most frequent users — patients with serious chronic illness. These patients must manage ongoing chronic diseases while also facing frequent acute care and challenges at the end of life. They are also at risk for an overmedicalized, burdensome, and depersonalized experience [5, 6]. If patient experience is truly valued, it is these patients who may require the most attention.

Patient experience is a standard health care measure, payment criterion, and pillar in the Triple Aim [2, 7]. Current industry standard patient experience tools deployed in the U.S., that are mandated by the ACA [4, 7, 8], such as general and setting-specific variations of the Consumer Assessment of Healthcare Providers and Systems (CAHPS; e.g., for hospitals, clinics, or hospice) focus on doctor-patient communication, access to care, and overall ratings of experience [9–11]. These tools largely ignore the broader context of care delivery via teams, and are not particularly useful in understanding the experiences of patients with serious chronic illness, as they encounter care across settings, health declines, and life transitions [12].

Many surveys of patient experience exist outside of the U.S. [13] but few focus on patients with serious illness, favoring a generalized approach to measurement. In England, the National Health Service has been collecting data on patients’ experience for over a decade [14]. Yet, to our knowledge, no national health survey tailored to patients with serious chronic illness exists in England. However, some countries are developing tools tailored to more advanced patient populations. The Ontario Hospital Association is developing a longitudinal patient experience tool designed to capture a wide set of experience measures for the complex continuing care sector [15]. Of the few tools that do exist for patients with serious illness, many have limitations.

Primarily, experience tools oriented toward palliative care or end-of-life populations are often delivered too late in the serious illness trajectory. Often the focus is within the last 6 months of life or post hoc instruments of the bereaved [11, 16, 17]. Many tools do not ask patients about medical and non-medical goals of care, care team relationships versus communication, or whether patients feel the care team understands the whole individual versus solely aspects of patients’ physical wellbeing.

To address these gaps, our objective was to develop a new patient experience measurement tool for individuals with serious chronic illness that could be administered longitudinally, as part of a larger health care delivery intervention, and evaluate its psychometric properties.

## Methods

### Study design and context

This is an observational study aimed at developing and validating a novel experience tool for individuals with serious chronic illness in later life. It is part of a larger evaluation of LifeCourse, a late-life care intervention, which enrolled patients from October 2012 to July 2016 at a large, not-for-profit, integrated health system in the upper Midwest. Allina Health has 13 hospitals, 84 primary care and hospital-based clinics, 15 retail pharmacy sites, and 2 ambulatory care centers throughout Minnesota and western Wisconsin. Patients were recruited after encounters at hospitals or clinics geographically centered in Minneapolis and Saint Paul, Minnesota or one of the nearby suburbs. Surveys were administered to patients quarterly beginning on the enrollment date and continuing until death or loss to follow-up.

### Scale development

We developed the scale in stages between May and September 2012. First, we conducted listening sessions organized by Twin Cities Public Television (TPT). Participants included stakeholders ranging from patients living with a life limiting illness and their key family and friends to clinicians and research team members. TPT recruited participants to discuss their experience with late life care as part of a planned documentary series on late life in Minnesota, http://www.tpt.org/late-life/. The sessions were filmed by TPT and facilitated by a marriage and family therapist who is also a research scientist on the team. Sessions were edited by TPT and then transcribed to inform intervention development. A workgroup of experts was convened including clinicians in palliative care and hospice and researchers in the areas of long-term care and aging, patient-centered outcomes, team-based interventions, and practice-based evaluation. They were primarily tasked with intervention design during this stage. A secondary focus on learning more about the experience of care for patients near the end of life emerged early on in the intervention design as one of many key outcomes to be evaluated. This stage helped to inform the team what matters most to patients and families.

Subsequently, a workgroup of clinicians and research team members was formed to make critical decisions on measurement of patient experience. The goal of the workgroup was to evaluate the efficacy of current experience surveys and their applicability to both our intervention design and patient population. We decided that the intervention needed a general use scale appropriate for our population that was agnostic of care setting and could be deployed longitudinally to be useful in a broad system-wide context. We then conducted a literature review to evaluate existing measures. No measure was found to be salient enough to our intervention or population of interest so the group decided to develop its own measure loosely based on the intervention’s guiding principles but not designed to measure them directly. Further, a patient and caregiver advisory council offered insight to the study team. Qualitative findings were used to help clarify the intervention’s guiding principles. The final set of guiding principles—Know Me, Ask Me, Listen to Me, Hear Me, Guide Me, Respect Me, Comfort Me, and Support Me—were used as a general framework for deriving the survey’s domains and identifying associated survey items of potential interest.

Finally, candidate items found via literature review of existing experience survey tools [10, 12, 18] in general and as related to palliative care, hospice, and other settings were reviewed in multidisciplinary team discussion focused on comparing items to guiding principles and LifeCourse intervention components (e.g., whole-person care, patient goals). In several cases, existing tools did not address LifeCourse’s guiding principles (e.g., ongoing interpersonal relationships with care team), and so the team crafted and reviewed its own items to address those aspects. Existing scales were referenced for formatting, layout, and overall design elements but not for item content. A candidate pool of 34 items was created and selected by workgroup members. We conducted a pilot study on 35 patients to informally evaluate the working scale. Refinements were made by the workgroup based on interviews with patients and feedback from trained research interviewers who conducted surveys in person. Redundant and confusing items were refined or removed based on cognitive debrief interviews and a health literacy assessment (REALM-R) of early versions of the tool among pilot participants. Interviews and assessments focused on the applicability of specific word choice, response options, and other issues [19]. Special attention was paid to the survey length, survey formatting, and ease of interpreting items due to the advanced age and illnesses present in the majority of the sample. Calibri 14 point font was used to increase readability for visually impaired participants and the tool scored 72.6 (7^th^ Grade) for Flesch Reading Ease and 5.9 for the Flesch-Kincaid Grade Level.

Following our development process, the LifeCourse experience tool tested in this paper included 25 items. All items focused on experience associated with the participants’ care team during the past 30 days and used a four-point, frequency-based adjectival scale (1 = “Never”; 2 = “Sometimes”; 3 = “Usually”; 4 = “Always”), except for items related to patients’ goals for their care, which used a Likert scale with agreement-based responses (1 = “Strongly Disagree”; 2 = “Disagree”; 3 = “Agree”; 4 = “Strongly Agree”). The care team was purposefully defined to be inclusive across care settings to reinforce a whole system approach and included all members of likely multiple care teams (e.g., physicians, nurses, aides, care guides, social workers, chaplains, and others). Two sets of items regarding experience during major transitions and needing additional services or resources had valid skip patterns (i.e., were structured missing) for individuals who did not have a transition or need additional services. These items were not included in the overall scale, though we did collect them for assessment of the intervention. Additionally, we did not include two global questions (“Rate your care over the past 30 days.”, “Rate your support over the past 30 days.”) in factor analyses due to their high correlations with nearly all domains and items. These two items were instead used in tests of construct validity with the overall scale.

### Participants

**Fig. 1:**
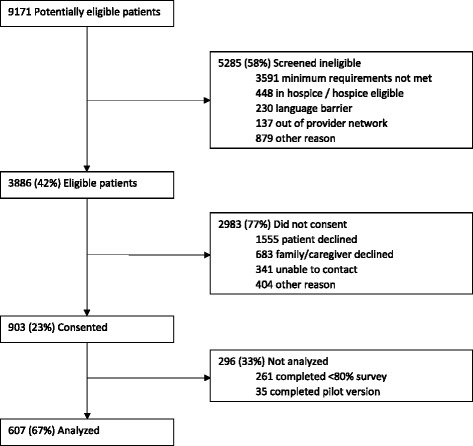
Patient eligibility screening, enrollment, and analysis flow

For this paper, we included a total of 903 enrolled patients in the analysis. Patients were identified as eligible for intervention and comparison groups through the combination of an electronic health record eligibility list – which listed emergency department and inpatient utilization in the prior year, advanced primary diagnosis of heart failure, cancer, or dementia, and a validated comorbidity index [20]. The eligibility list risk stratified patients based on their comorbidity score. For patients with dementia there was no comorbidity cutoff. For all other patients a comorbidity score of 4 or greater was required. A confirmative chart review was conducted by an experienced registered nurse. Selected patients were not yet hospice-eligible but sick enough to receive benefits of a supportive care approach 2 to 3 years prior to death. Detailed information about hospice eligibility criteria used can be found in Additional file 1. Also, 35 patients were excluded for this study because they had received an early pilot version of the LifeCourse experience tool. We excluded 261 patients who had completed fewer than 80% of items, leaving 607 patients with data for subsequent analyses (Fig. 1).

### Analyses

While we designed the experience tool as a complete scale, we also tested the usefulness of specific domains as independent subscales. We examined internal consistency, test-retest reliability, item correlations, and a random split-half cross validation design using exploratory and confirmatory factor analyses. We used baseline measures for the factor analyses and baseline through 3-month responses to calculate intraclass correlation for test-retest reliability of the scale. All analyses were conducted in Stata/MP version 14.1 [21].

We used exploratory factor analysis (EFA) to identify a measurement model in the development cohort and then subsequently evaluated factors/domains using confirmatory factor analysis (CFA) in the validation cohort [22]. EFA used principal factor estimation and oblique (promax) rotation. We determined the optimal number of factors using the Kaiser criterion (i.e., eigenvalues > 1) and a scree plot [23]. In a scree plot, eigenvalues are plotted in descending value. The last substantial drop was present between an eigenvalue of 3 and 4 prior to flattening out suggesting we retain 3 factors [24]. Both the Kaiser criterion and the scree plot suggested we choose a 3 factor model.

We conducted CFA of the experience subscales using full information maximum likelihood [25, 26]. Factor loadings from the EFA that were higher than 0.35 were freely estimated while the rest were fixed at 0. Correlations between latent factors were freely estimated as well, and only the ones that significantly improved the fit of the model and that were statistically significant were retained. Model goodness of fit was evaluated using fit indices available in Stata, including the comparative fit index (CFI), the Tucker-Lewis index (TLI), and the root mean square error of approximation (RMSEA) [27].

We used Cronbach’s alpha to assess individual domain and overall scale internal reliabilities of the final scale with 0.80 considered as sufficient internal reliability. The intraclass correlation coefficient (ICC) was calculated on the total score using an unadjusted mixed-effects linear model on a subsample of 360 participants’ baseline and 3 month measurement to assess test-retest reliability. To assess general construct validity, we correlated the overall score with two global items asking patients to “Rate your care” and “Rate your support”.

## Results

### Analytic sample

On average, respondents were aged 74 years, 50% were female, and had 5 comorbidities. The majority of the respondents were living at home with a primary diagnosis of advanced heart failure. Participants had 5 inpatient days, 2 emergency department visits, and 1 intensive care unit stay in the 12 months prior to selection. The randomly split development and validation cohorts had 304 and 303 cases, respectively. Patient characteristics were not found to be statistically different between the development and validation samples (Table 1).

**Table 1:** Sample description overall and by cohort

Characteristic	Overall (*N* = 607)		Development Cohort (*N* = 304)		Validation Cohort (*N* = 303)		*P*-Value^a^
	*N*	Mean ± SD/%	*N*	Mean ± SD/%	*N*	Mean ± SD/%	
Age, years	607	74 ± 13	304	74 ± 12	303	74 ± 13	0.915
Comorbidity Score^b^	564	5 ± 2	280	5 ± 2	284	5 ± 2	0.745
Utilization^c^							
ED Visits	607	2 ± 2	304	2 ± 2	303	2 ± 3	0.963
Inpatient Days	607	5 ± 6	304	4 ± 6	303	5 ± 6	0.926
ICU Days	607	1 ± 3	304	0 ± 3	303	1 ± 4	0.285
Male	306	50%	149	49%	157	52%	0.490
Married or Living with Partner	286	47%	140	46%	146	48%	0.599
Caucasian	576	95%	291	96%	285	94%	0.352
Highest Level of Education							0.232
Non-Graduate, H.S. or GED	189	31%	99	33%	90	30%	
Some College to 4-year Graduate	283	47%	148	49%	135	45%	
Graduate or Professional School	123	20%	52	17%	71	23%	
Unknown	12	2%	5	2%	7	2%	
Participant Location at Baseline							0.539
Home	528	87%	269	88%	259	85%	
Assisted Living	58	10%	26	9%	32	11%	
Nursing Home	21	3%	9	3%	12	4%	
Primary Diagnosis							0.485
Heart Failure	426	70%	220	72%	206	68%	
Cancer	118	19%	54	18%	64	21%	
Dementia	63	10%	30	10%	33	11%	

^a^Chi-square tests were used for categorical variables and two-sample t-tests for continuous variables

^b^43 participants were missing a baseline comorbidity score

^c^Health care utilization measures were estimated in the 12 months prior to selection

### Exploratory factor analysis

The EFA suggested a 3-factor model which accounted for 92% of the total item variances (63%, 16%, and 13%). Item 30, “My problem or physical symptom was well controlled”, and item 9, “I received conflicting advice from members of my care team”, were removed since they did not load ≥0.35. Additionally, item 32, “I was frustrated by the care I received”, cross-loaded on two factors and was removed. We extracted three subscales from the 22 remaining items: Care Team (14 items); Communication (5 items); and Care Goals (3 items). For all factors, we found good loadings for almost all items; all loadings were between 0.40 and 0.84 (and most above 0.50). Items with rotated factor loadings of the domains can be found in Table 2.

**Table 2:** Rotated factor loadings from exploratory factor analysis in the development cohort (*N* = 304)

Items	Factor 1	Factor 2	Factor 3
Chronbach’s Alpha	0.897	0.811	0.821
The care team helped me make a choice about my care when I had one. (Q12)	0.742	−0.029	0.056
The care team kept my wishes at the center of my care. (Q14)	0.737	0.012	−0.025
The care team helped me understand all of my options when I had a choice about my care. (Q11)	0.722	0.070	0.025
The care team respected me. (Q13)	0.709	0.010	−0.073
The care team helped me understand what was important to me. (Q10)	0.676	−0.038	0.075
I trusted my care team. (Q33)	0.650	0.015	−0.054
The care team helped me determine which providers I needed to see. (Q15)	0.634	−0.113	−0.008
The care team spent the right amount of time with me. (Q17)	0.630	0.075	−0.034
I received easy to understand information from the care team in response to my questions. (Q8)	0.624	0.034	0.044
The care team did everything they could to help with my problem or physical symptom. (Q31)	0.613	0.042	0.076
I was able to get in touch with someone on my care team when needed. (Q18)	0.568	0.117	−0.102
I got appointments as soon as I needed them. (Q16)	0.463	−0.006	0.002
The care team knew my personal circumstances or situation. (Q5)	0.458	0.048	0.131
The care team relied upon my ideas to manage my care. (Q4)	0.403	−0.117	0.122
I had to repeat myself when telling the care team about my life. (Q3)	−0.035	0.750	0.033
I had to repeat myself when telling the care team about my medical condition. (Q1)	−0.018	0.712	−0.054
I had to repeat myself when telling the care team about what was important to me. (Q2)	0.137	0.702	−0.081
I had unanswered questions about how my illness affected my everyday life. (Q7)	−0.017	0.671	0.074
I had unanswered questions about how my illness affected my health. (Q6)	−0.050	0.649	0.051
I have good understanding of my goals of care. (Q28)	0.003	0.062	0.841
I know what I need to do as part of my goals of care. (Q29)	0.013	0.012	0.807
My goals of care include what is important to me. (Q27)	0.051	−0.044	0.648

### Confirmatory factor analysis

The 3-factor model from the EFA in the development cohort was used as the initial measurement model for the CFA and tested in the validation cohort. The initial model exhibited poor fit *X* ^2^ (206) = 565.37, *p* < 0.001; CFI = 0.879; TLI = 0.864; RMSEA = 0.076. To address the lack of fit, we explored model respecification as an iterative process using a combination of the modification indices (MI), expected parameter change (EPC), and theoretical plausibility. Goodness-of-fit statistics after each respecification can be found in Table 3.

**Table 3:** Fit indices for each model tested during confirmatory factor analysis in the validation cohort (*N* = 303)

Modeling Step	*X* ^2^	*df*	*P*	CFI	TLI	RMSEA
1. Initial model suggested by EFA	565.372	206	<0.001	0.879	0.864	0.076
2. Removed item 4 for weak loading (0.33)	524.304	186	<0.001	0.884	0.869	0.077
3. Added path between error terms for items with similar wording and adjacency	358.480	179	<0.001	0.939	0.928	0.058
4. Added path between error terms for care team items related to access	327.332	176	<0.001	0.948	0.938	0.053
5. Added path between error terms for care team items related to care delivery	295.626	173	<0.001	0.958	0.949	0.048

*EFA* Exploratory Factor Analysis, *CFI* Comparative Fit Index, *TLI* Tucker - Lewis Index, *RMSEA* Root Mean Square Error of Approximation

After examining the model coefficients we discovered item 4, “The care team relied upon my ideas to manage my care”, did not load ≥ 0.35. This item exhibited weak loadings in both the development and validation cohort, possibly indicating it is associated with another unmeasured factor so it was removed from analysis. Subsequently, all estimated coefficients in the model indicated that the parameters from all three latent variables to each of their items were all statistically significant with fair to strong loadings (0.50–0.86), indicating that the items related to their factors.

MI and EPC suggested that there were relationships among some of the residuals between items within factors. After closely examining the items, we allowed theoretically plausible correlations between some of the residuals within the scale, which improved model fit. These correlations were based on method effects which we grouped into categories: (1) items which share similar wording and adjacent to each other on the questionnaire, (2) care team related items which address access to care, and (3) care team related items which address care delivery. Error terms for these items were allowed to be freely estimated and each model improved the fit significantly (Table 3).

**Fig. 2:**
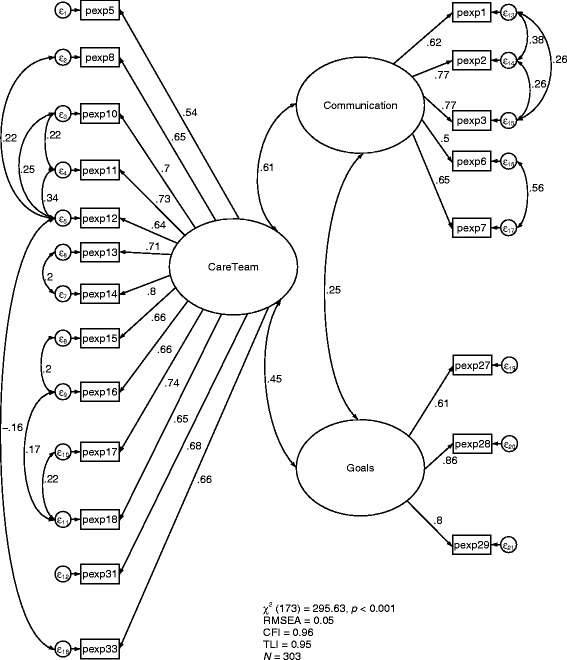
Final confirmatory factor analysis (CFA) model of the LifeCourse patient experience tool (*n* = 303)

Factor loadings, factor labels, and correlations among factors for the final model are presented in Fig. 2. The final model exhibited good fit as evidenced by the Chi-square/*df* ratio (1.71) below 2; *X* ^2^ (173) = 295.63, *p* < 0.001; CFI = 0.958; TLI = 0.949; RMSEA = 0.048. Weak to moderate intercorrelations between domains were present. The correlation between Care Team and Communication was 0.61, Care Team and Care Goals was 0.45, and Communication and Care Goals was 0.25.

Internal reliabilities were checked by calculating Cronbach’s alpha for each domain and the entire scale (Care Team = 0.92, Communication = 0.83, Care Goals = 0.77, LifeCourse Experience Scale = 0.91). Test-retest reliability for the LifeCourse experience scale was measured among participants surveyed at baseline and 3 months (ICC = 0.58; 95% CI: 0.52–0.65). Since our experience tool is designed to be deployed longitudinally, a high test-retest reliability is not desirable for quarterly intervals as it would limit the ability of a tool administered at multiple time points to detect change. Construct validity was measured through correlating the LifeCourse Experience Scale with two global items which asked participants to “Rate your care” and “Rate your support” over the past 30 days. Both items correlated significantly with the scale, r = 0.65 and r = 0.64, respectively. Since these items correlated highly with all domains and nearly all items, they were used as indicators for a broad gauge of the construct of patient care experience.

## Discussion

A newly developed patient experience scale demonstrated high reliability and validity and could be used in further evaluation of care delivery experiences for those late in life. Experience tools oriented toward existing relationships and later-life care for complex patients may allow for meaningful assessment and better understanding of targets for integrating and streamlining care. This is especially important since existing patient-centered tools, such as decision aids, do not guarantee that patients will be treated as partners or that providers will understand their wishes in late life [28, 29]. New models of care, regardless of their design, require tracking and assessment with regard to their impact on relationships and communication with care teams and whether patients’ goals are actively understood. This is particularly true in later life. As patients age, their health needs increase exceeding their individual capacity [30].

Several strengths in the design of this scale improve measurement of patient experience in patients with chronic life-limiting illnesses. First, our scale asks about care teams as a whole unlike many of the standard experience scales which focus on doctor or nurse-only experience. As health systems restructure toward new care delivery models oriented on team-based care, focus on evaluating teams is increasingly important. The care team domain we designed also seeks to address interpersonal aspects of experiences between patients and care teams. Second, our tool was designed to be implemented across settings with a broad focus and targets patients earlier in the chronic illness trajectory than existing scales. For patients frequently experiencing complex health care interactions in siloed health system divisions, this is perhaps a more realistic reflection of what patients experience. Finally, our scale assesses patients’ goals of care. In a time of increasingly difficult care decisions which often carry heavy consequences for patients and their families, health systems need to focus more on patient-defined medical and non-medical goals in their efforts to improve care.

The usefulness of this tool may also be understood within a global context in which chronic and non-communicable diseases account for nearly 90% of deaths in high-income countries (and increasing to nearly 70% worldwide by 2030) [31]. Work on multimorbidity and patient burden in chronic conditions such as heart failure in Europe, including guidelines on multimorbidity and measurement of treatment burden [6, 32–34], reflects recognition of the patient-facing side of this reality. In such a context, understanding how to address individuals’ needs via integrated and holistic palliative care services is vital, yet one review found only 20 countries to have advanced levels of palliative care integration (about a third of countries worldwide had no known hospice or palliative care activity) [35]. Among countries working toward palliative care integration, standardization and an evidence base derived from rigorous, patient-centered assessment remain the focus of existing frameworks and calls for further work [36–38]. In this context, we believe that an experience tool for patients with serious chronic conditions in later life may help to form such an evidence base to help drive practice standards and improve care.

### Limitations

This study has several limitations. Factor loadings could be artificially inflated due in part to either the similarity in item wording, likeness of the topic, and/or the adjacency of items concerning the same topic in the survey. However, the fact that items with disparate wording also loaded together on the same factors (e.g., in the Communication domain) suggests that the underlying constructs were relatively cohesive across item content. Correlated error terms could be indicative of underlying undefined factors in the Care Team and Communication domains or due to simple design effects such as clustering of questions about similar topics on the survey. Item and questionnaire refinement focused on care team access and the practical and interpersonal aspects of care delivery, followed by additional analyses, could address this limitation. However, this would need to be balanced against additional length in a survey of patients already prone to high rates of missing data.

With regard to item and unit missingness, future work should focus on the recall time frame — as a 30 day lookback period may be too brief if patients, despite having complex conditions, are not having frequent visits. Conversely, it is also possible that respondents who have had multiple encounters of various quality over longer lookback periods struggle to average their experience as a whole. There is a limited amount of research about the ability of patients to recall their previous experience and how it may be affected by reference periods [39–41]. Alternative event-based approaches, such as that used by H-CAHPS [10] may address this issue. However, there are tools currently in large scale use in U.S. clinic settings with longer lookback periods, like the 12 month period used in the CG-CAHPS [9, 42] tool, which may exacerbate recall issues.

Further, encounter-based assessment may sacrifice relational aspects of experience regarding care teams. The CAHPS suite of tools assume a lot about the patient-provider and patient-team relationship that is unlikely to fit patients who see a lot of providers and overlooks the team-based approach entirely. H-CAHPS and hospice CAHPS [11] are event driven and hospice CAHPS is sent to caregivers after the patient has died, so the measures don’t capture the patient’s overall, ongoing relationship with a team across a number of settings and events like our tool. In addition, CAHPS experience tools administered in older populations also suffer from lower response rates [43, 44]. Additional validity studies in different samples drawn at different times are needed to ensure consistency of the results reported here. Furthermore, our sample may not be representative of the population at large and should be replicated in samples with more diverse demographic profiles.

Missing data were somewhat problematic in our sample — a common issue with survey burden in studies of patients with advanced illness [45, 46]. We addressed this in analyses in two ways. First, patients with <80% response (i.e., ≥5 missing items out of 25 total items) were dropped from the analysis. Second, we used maximum likelihood with the expectation-maximization (EM) algorithm to estimate the covariance matrix [47], the EM covariance matrix was used to obtain a factor solution. A factor loading cutoff of 0.35 was used for low loadings or cross-loadings (cases with high loading on more than one factor) [48].

## Conclusions

With its focus on care teams, communication, and care goals for patients with serious chronic illness, our new experience tool and its subscales, display strong reliability and perform well psychometrically. This LifeCourse experience tool, while developed as part of an intervention study, may prove highly useful in describing and studying patient experience across this and other populations, helping to further establish experience as a core component of care quality and value for all patients served by healthcare.

## Additional file

Additional file 1: Eligibility criteria for hospice. (PDF 53 kb)

## Abbreviations

ACA: Affordable Care Act
CAHPS: Consumer Assessment of Healthcare Providers and Systems
CFA: Confirmatory factor analysis
CFI: Comparative fit index
CMS: Centers for Medicare & Medicaid Services
EFA: Exploratory factor analysis
EM: Expectation-maximization
EPC: Expected parameter change
MI: Modification index
RMSEA: Root mean square error of approximation
TLI: Tucker-Lewis index
TPT: Twin Cities Public Television
