# Supplementary material for: Development and validation of a new patient experience tool in patients with serious illness
Source: BMC Palliat Care. 2016 Dec 30;15:99. doi: 10.1186/s12904-016-0172-x (PMC5203706; doi:10.1186/s12904-016-0172-x)
Supplement: Additional file 1: — Eligibility criteria for hospice. (PDF 53 kb) [file 12904_2016_172_MOESM1_ESM.pdf]

Allina Health
HOSPICE & PALLIATIVE CARE

Eligibility criteria for hospice

Diagnosis	Main criteria<br>(generally these clinical indicators are present)	Supporting criteria	Comments
General guidelines	1. Life-limiting condition; estimated prognosis of six months or less. ***"Is this patient sick enough to die in the next 6 months?"***<br>2. Patient and/or family have elected treatment of symptoms, not cure of underlying disease<br>3. One of the following:<br>♦ Clinical progression of disease<br>♦ Impaired nutrition related to the terminal process with weight loss	♦ Multiple ER visits or hospitalizations<br>♦ Dependence in 2 or more ADLs (bathing, dressing, feeding, transfers, continence, independent ambulation)<br>♦ Karnovsky < 70%<br>♦ Unintentional 10% weight loss over 6 months<br>♦ Albumin < 2.5	Co-morbid conditions, the severity of which is likely to contribute to a life expectancy of 6 months or less should be considered.
Heart disease	1. NYHA Class IV (symptoms of dyspnea or angina virtually at rest)<br>2. Optimal medical treatment<br>3. Not a candidate for transplant<br>4. Pump failure	♦ Resistant arrhythmias<br>♦ History of cardiac arrest<br>♦ Unexplained syncope<br>♦ HIV disease<br>♦ Symptomatic with any physical activity	EF 20% or less is helpful objective evidence HX of cardiac arrest, resuscitation, unexplained syncope or brain embolus of cardiac origin could contribute to eligibility.
Pulmonary disease	1. Disabling dyspnea at rest with decreased functional ability<br>2. Disease progression (increased hospitalizations, pulm, infections, respiratory failures)<br>3. Serial decrease FEV1>40ml/yr (not nec. to obtain)	♦ Cor pulmonale or right heart failure<br>♦ Rest pO_2 <= 55 mm<br>♦ SaO_2 <= 0.88 on supplemental O_2<br>♦ PCO_2 =>50 mm<br>♦ Resting tachycardia > 100<br>♦ Progressive weight loss	FEV_1 < 30% predicted after bronchodilator therapy.
Dementia	1. Dependence in *all* ADLs (ambulating, dressing, bathing, urine/stool continence)<br>2. No meaningful verbal communication (6 or fewer words)<br>3. Presence of a medical complication in past year<br>4. FAST assessment of <=7	Medical complications (e.g.):<br>♦ Aspiration pneumonia<br>♦ Upper UTI/pyelonephritis<br>♦ Sepsis<br>♦ Stage 3-4 pressure ulcers<br>♦ Recurrent fever after antibiotics	Inability to maintain sufficient fluid and calorie intake or serum albumin of <2.5gm/dl also indicate decline.
Liver disease	1. Severely impaired liver function (albumin < 2.5, prolonged INR >1.5, prothrombin >5 sec)<br>2. One of the following: ascites, hepatorenal syndrome, hepatic encephalopathy, recurrent variceal bleeding, bacterial peritonitis	♦ Progressive malnutrition, muscle wasting<br>♦ Active drinking<br>♦ Hepatocellular carcinoma<br>♦ Hepatitis B<br>♦ Hepatitis C refractory to interferon tx	

S412798 © 2014 ALLINA HEALTH SYSTEM ® A REGISTERED TRADEMARK OF ALLINA HEALTH SYSTEM

Diagnosis	Main criteria<br>**(generally these clinical indicators are present)**	Supporting criteria	Comments
Renal disease	1. Creatinine clearance < 10cc/min (<15 cc/min for diabetes), GFR <10ml/min<br>2. Creatinine > 8 (>6 for diabetics)<br>3. Signs and symptoms: uremic syndrome, oliguria, intractable hyperkalemia, pericarditis, intractable fluid overload<br>4. Not seeking dialysis or transplant, or discontinuing dialysis	♦ Mechanical ventilation<br>♦ Cancer<br>♦ COPD<br>♦ CHF<br>♦ Liver disease<br>♦ Sepsis<br>♦ Albumin < 3.5, PLT < 25,000, Age > 75	Dialysis does not preclude eligibility for hospice but continuation with life extension beyond 6 months would impact eligibility.<br><br>Another diagnosis for terminal status could be appropriate with the continuation of dialysis.
Stroke and coma	1. Coma/vegetative state persisting beyond 3 days' duration<br>2. Karnofsky or palliative performance scale of <40.<br>3. Dysphagia severe enough to prevent continuation of fluids/food necessary to sustain life without artificial nutrition.	♦ Absent verbal response<br>♦ Absent pain withdrawal<br>♦ Creatinine > 1.5<br>♦ Age > 70<br>♦ Abnormal brain stem response<br>♦ Diagnostic imaging which support poor prognosis	Additionally, documentation in the previous 12 months of aspiration pneumonia, pyelonephritis, stage 3 or 4 decubitis, recurrent fever after antibiotics.
Amyotrophic Lateral Sclerosis	1. Rapid progression (most of disability developing within the last 12 months)<br>**AND**<br>2. One of the following:<br>♦ Critically impaired ventilatory capacity / FVC < 30% predicted<br>♦ Critical nutritional impairment<br>♦ Life-threatening complication	Complications:<br>♦ Aspiration pneumonia<br>♦ Upper UTI<br>♦ Sepsis<br>♦ Stage 3-4 pressure ulcers<br>♦ Recurrent fever after antibiotics	The decision to institute either artificial ventilation or artificial feeding may significantly alter 6 month prognosis.<br><br>Neurologist exam within 3 months of hospice assessment is recommended to confirm prognosis.
HIV disease	1. Viral load > 100,000<br>**OR**<br>2. Lower viral load, but foregoing ARV and prophylactic therapy<br>3. CD4+ count <25 when patient is free of acute illness<br>4. Karnofsky scale at <=50	♦ CNS lymphoma<br>♦ Wasting syndrome (33% of lean body mass)<br>♦ Kaposi's involving viscera, unresponsive to therapy<br>♦ Renal failure<br>♦ Advanced dementia, PML<br>♦ Toxo, crypto, MAC bacteremia, untreated	Albumin < 2.5<br>Ongoing substance abuse<br>Age > 50<br>CHF, dilated cardiomyopathy<br>Chronic persistent diarrhea >1 year
Cancer	1. Disease with metastases or progression from an earlier stage of disease to metastatic disease	♦ Continued decline in spite of therapy<br>♦ Patient declines further disease directed therapy.	Certain cancers with poor prognosis (e.g. small cell lung cancer, brain cancer and pancreatic cancer) may be hospice eligible without other eligibility criteria listed.

These guidelines were developed from the LCD-determining terminal illness guidelines provided by CMS.
For further information related to hospice care, call the Allina Health Care Navigation Help Desk at 612-262-2200.

S412798 © 2014 ALLINA HEALTH SYSTEM ® A REGISTERED TRADEMARK OF ALLINA HEALTH SYSTEM
